# CD19-immunoPET for noninvasive visualization of CD19 expression in B-cell lymphoma patients

**DOI:** 10.1186/s40364-024-00595-9

**Published:** 2024-05-12

**Authors:** Dominik Sonanini, Johannes Schwenck, Simone Blaess, Julia Schmitt, Andreas Maurer, Walter Ehrlichmann, Malte Ritter, Julia Skokowa, Manfred Kneilling, Gundram Jung, Falko Fend, Simon Krost, Christian M. Seitz, Peter Lang, Gerald Reischl, Rupert Handgretinger, Christian la Fougère, Bernd J. Pichler

**Affiliations:** 1 Werner Siemens Imaging Center, Department of Preclinical Imaging and Radiopharmacy, University of Tübingen, Röntgenweg 13, 72076 Tübingen, Germany; 2 Department of Medical Oncology and Pneumology, University Hospital Tübingen, University of Tübingen, Tübingen, Germany; 3 Cluster of Excellence iFIT (EXC 2180) “Image-Guided and Functionally Instructed Tumor Therapies”, University of Tübingen, Tübingen, Germany; 4 Department of Nuclear Medicine and Clinical Molecular Imaging, Department of Radiology, University of Tübingen, Tübingen, Germany; 5 German Cancer Consortium (DKTK) and German Research Center (DKFZ), Partner Site, Tübingen, Germany; 6 Department of Hematology, Oncology, Clinical Immunology and Rheumatology, University Hospital Tübingen, University of Tübingen, Tübingen, Germany; 7 Department of Dermatology, University of Tübingen, Tübingen, Germany; 8 Interfaculty Institute for Cell Biology, Department of Immunology, University of Tübingen, Tübingen, Germany; 9 Institute of Pathology and Neuropathology, University Hospital Tübingen, University of Tübingen, Tübingen, Germany; 10 University Children’s Hospital, University of Tübingen, Tübingen, Germany

**Keywords:** CD19, Positron emission tomography, Imaging biomarker, Theranostics, CAR-T cells, Molecular imaging, B-cell non-hodgkin lymphoma

## Abstract

Cell- and antibody-based CD19-directed therapies have demonstrated great potential for treating B-cell non-Hodgkin lymphoma (B-NHL). However, all these approaches suffer from limited response rates and considerable toxicity. Until now, therapy decisions have been routinely based on histopathological CD19 staining of a single lesion at initial diagnosis or relapse, disregarding heterogeneity and temporal alterations in antigen expression. To visualize in vivo CD19 expression noninvasively, we radiolabeled anti-human CD19 monoclonal antibodies with copper-64 (^64^Cu-αCD19) for positron emission tomography (CD19-immunoPET). ^64^Cu-αCD19 specifically bound to subcutaneous Daudi xenograft mouse models in vivo. Importantly, ^64^Cu-αCD19 did not affect the anti-lymphoma cytotoxicity of CD19 CAR-T cells in vitro. Following our preclinical validation, ^64^Cu-αCD19 was injected into four patients with follicular lymphoma, diffuse large B-cell lymphoma or mantle zone lymphoma. We observed varying ^64^Cu-αCD19 PET uptake patterns at different lymphoma sites, both within and among patients, correlating with ex vivo immunohistochemical CD19 expression. Moreover, one patient exhibited enhanced uptake in the spleen compared to that in patients with prior B-cell-depleting therapy, indicating that ^64^Cu-αCD19 is applicable for identifying B-cell-rich organs. In conclusion, we demonstrated the specific targeting and visualization of CD19^+^ B-NHL in mice and humans by CD19-immunoPET. The intra- and interindividual heterogeneous ^64^Cu-αCD19 uptake patterns of lymphoma lesions indicate variability in CD19 expression, suggesting the potential of CD19-immunoPET as a novel tool to guide CD19-directed therapies.

**Supplementary Information:**

The online version contains supplementary material available at 10.1186/s40364-024-00595-9.

To the editor

CD19-directed therapies, such as chimeric antigen receptor (CAR)-T cells, the Fc receptor-optimized monoclonal antibody (mAb) Tafasitamab-cxix or the mAb-drug conjugate Loncastuximab tesirine, have emerged as relevant treatment alternatives for B-cell non-Hodgkin lymphoma (B-NHL). While some patients achieve complete and durable remission, the overall response rate of ∼ 50% falls considerably below that observed in Pro-B-ALL patients. Furthermore, 70% of B-NHL patients fail to achieve long-term survival, exposing them to significant toxicity, particularly neurotoxicity or cytokine release syndrome [1–5].

In contrast to single-cell leukemia targeting, challenges such as impaired lymphoma cell accessibility, a complex immunosuppressive microenvironment, inter- and intraindividual alterations in CD19 expression, CD19 epitope loss or downregulation following CD19-directed therapy, and unreliable immunohistochemical CD19 staining limit accurate patient stratification and therapeutic success in B-NHL patients [6–9]. Beyond histopathological assessment, there is currently no target-specific approach available that can be used for patient stratification and treatment decision-making in B-NHL.

Positron emission tomography (PET) with radiolabeled antibodies (immunoPET) enables whole-body visualization and quantification of specific target expression over time, therapeutic drug biodistribution, and tumor accessibility [10]. Recently, we revealed heterogeneous GD2-derived uptake patterns and distinct alterations during targeted therapy in pediatric patients with metastatic neuroblastoma and sarcoma using a radiolabeled anti-GD2-mAb [11, 12]. In this study, we developed a copper-64 (^64^Cu)-radiolabeled mAb directed against human CD19 for positron emission tomography (PET) imaging and demonstrated, for the first time, the specific in vivo targeting and noninvasive visualization of CD19^+^ lymphoma lesions in experimental lymphoma-bearing mice and four human B-NHL subjects.

Radiolabeling of the αCD19-mAb (^64^Cu-αCD19) yielded a stable radioimmunoconjugate with minimal dimerization, high radiochemical (> 95%) and radionuclidic purity (≥ 99.9%), and an immunoreactivity of 57% (Fig. S1a, b). In vivo PET/MR and ex vivo biodistribution demonstrated significantly greater ^64^Cu-αCD19 uptake in subcutaneous Daudi lymphoma xenografts compared to a ^64^Cu-αB7-H3 control tracer (Fig. 1a, b; Fig. S1c). Importantly, the αCD19-mAb impaired αCD19-CAR-T-cell-mediated cytotoxicity in vitro only at concentrations ∼ 1000 times greater than the applied dose for PET imaging (Fig. 1c).

First-in-human PET/MRI scans were conducted ∼ 24 h after ^64^Cu-αCD19 injection in four B-NHL patients to evaluate eligibility for CD19-directed therapies (Fig. 2). As expected from previous therapeutic applications in childhood B-ALL patients treated with substantially higher mAb doses, all patients tolerated the ^64^Cu-αCD19 injections without any obvious clinical signs of toxicity.

Patient 1, with double-hit follicular lymphoma, exhibited remarkable tracer uptake in the cervical, abdominal, and singular bone lymphoma manifestations (Fig. 2a-c; average standardized uptake value, SUVavg 7.7–8.5). Interestingly, all lesions could be better differentiated by CD19-immunoPET than by [^18^F]FDG-PET/CT conducted 90 days before (Fig. S2a-c). Immunohistochemistry of a previously extirpated cervical lymph node revealed moderate CD19 protein expression. In contrast, the abdominal lymphoma bulk, irradiated by a total fractionated dose of 30 Gy with palliative intent shortly before, yielded little tracer accumulation (Fig. S2b; SUVavg < 1.5), suggesting residual necrotic/avital tissue.

In Patient 2, who suffered from refractory DLBCL, the thoracic (Fig. 2d) and abdominal lymphoma manifestations (Fig. 2d-f) indicated the strongest ^64^Cu-αCD19 accumulation among all the subjects (SUVavg up to 27.7). Likewise, intense histological CD19 expression was found in the resectate of a peritoneal lymphoma conglomerate. Furthermore, we detected markedly greater tracer uptake in the spleen (SUVavg 21.7) than in the other three patients (SUVavg 5.1–8.5). Notably, Patient 2 was the only subject who did not receive the B-cell-depleting αCD20-mAb rituximab within the last 6 months prior to CD19-immunoPET, indicating that ^64^Cu-αCD19 is applicable for detecting physiological B cells in lymphatic organs.

We further revealed pronounced tracer accumulation in the bone marrow of Patient 3 with DLBCL (Fig. 2h) compared to the other B-NHL patients (SUVavg 12.7 vs. 5.0, Patient 1). Interestingly, a subsequent bone marrow biopsy showed 99% B-NHL infiltration with intense CD19 expression, demonstrating the ability of CD19-ImmunoPET to differentiate CD19^+^ lymphoma lesions. However, we detected faint uptake in the known retrosternal and iliacal lesions (Fig. 2g, i) of Patient 3, which were highly suspicious of vital lymphoma according to [^18^F]FDG-PET/CT (Fig. S5d, f).

Moreover, in line with the low histological CD19 expression in an extirpated cervical lymph node from initial diagnosis, Patient 4, with marginal zone lymphoma, exhibited slight ^64^Cu-αCD19 uptake in the margin of a large pulmonary lymphoma bulk (Fig. 2j) and no relevant PET-derived signal in the retroperitoneal manifestations (Fig. 2l).

In conclusion, for the first time, we demonstrated the feasibility of detecting CD19^+^ lymphoma lesions noninvasively by CD19-immunoPET in B-NHL patients, which is fully consistent with the findings of our preclinical mouse studies. This innovative imaging approach may improve both patient stratification and therapeutic surveillance by detecting heterogeneous CD19 expression or antigen downregulation during CD19-directed therapies.

Furthermore, the noninvasive visualization of endogenous B cells holds great promise in multiple sclerosis [13] or for targeting tumor-associated tertiary lymphatic structures [14]. Finally, prospective clinical studies are needed to validate the optimal tracer dose, the accuracy of ^64^Cu-αCD19 uptake, the influence of physiological B cells on the PET uptake, potential tracer-related toxicities, and the superiority of this approach over conventional CD19 histopathology.

**Fig. 1:**
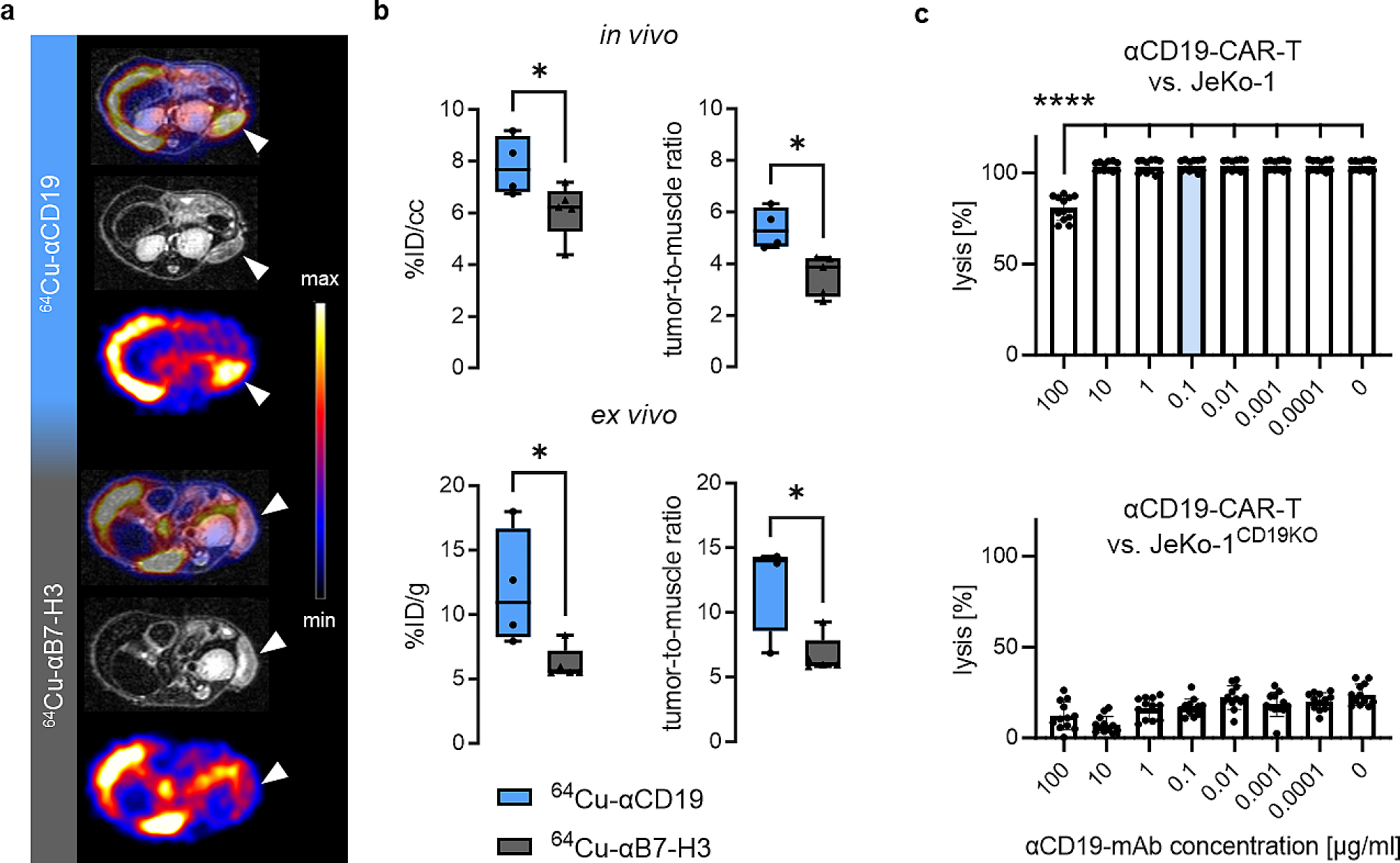
Preclinical evaluation of ^64^Cu-αCD19 and potential epitope blocking. (**a**) Representative transversal PET/MR (fused) as well as single MR and PET images of CD1 nude mice *subcutaneously* injected with Daudi lymphomas 48 h post-*i.v.* administration of ^64^Cu-αCD19 or unrelated isotype control (^64^Cu-αB7-H3). Lymphoma sites are marked by white arrows. (**b**) PET quantification of lymphoma uptake calculated as %ID/cc (in vivo) or %ID/g (ex vivo) and tumor-to-muscle ratios (*n* = 4–5 per group, unpaired t test, *P* values < 0.05 (*) were considered statistically significant). (**c**) Potential epitope blocking by αCD19-mAb and consecutive functional impairment of αCD19-CAR-T cells were tested in cytotoxicity assays against the CD19-expressing NHL cell line JeKo-1. αCD19-mAb dose titration demonstrated functional blocking effects at a concentration of 100 µg/ml (upper blot). CD19KO lymphoma cells served as a control to exclude target-independent effects (lower blot). (*n* = 12 per concentration, ordinary ANOVA, corrected for multiple comparison using the Tukey test, (****) *P* < 0.0001). The concentration of 0.1 µg/ml (blue) was calculated as the blood and lymphoma lesions based on the clinical PET/MR data

**Fig. 2:**
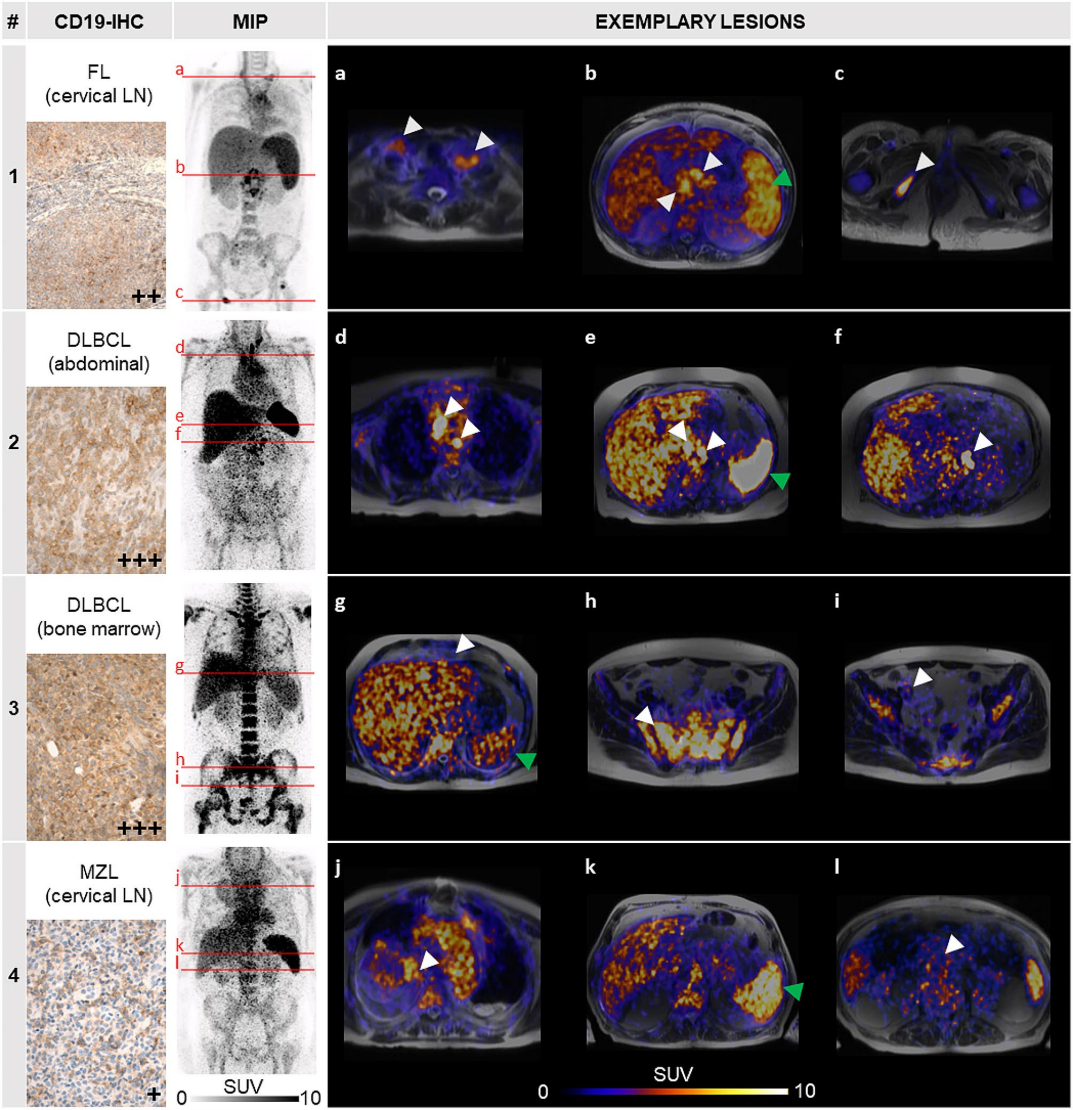
First-in-human application of ^64^Cu-αCD19. CD19-immunoPET was performed on four lymphoma patients [1–4] with different histological subtypes 19–25 h after ^64^Cu-αCD19 injection. Left: Immunohistochemical analysis of CD19 protein expression (CD19-IHC) in lymphoma tissues and maximum intensity projection (MIP) of standardized uptake values (SUV) for each patient. FL = follicular lymphoma, DLBCL = diffuse large B-cell lymphoma, MZL = mantle zone lymphoma, LN = lymph node. **a-l.** Exemplary transversal images (levels are marked in the MIP by red lines) of different lymphoma lesions (indicated by white arrows) and spleens (green arrow) are shown for each patient

### Electronic supplementary material

Below is the link to the electronic supplementary material.

Supplementary Material 1

## Data Availability

For original data, please contact Dominik.Sonanini@med.uni-tuebingen.de (preclinical data) or Christian.laFougere@med.uni-tuebingen.de (clinical data).

## Abbreviations

^64^Cu: copper-64
B-NHL: B-cell non-Hodgkin lymphoma
CD19: cluster of differentiation 19
CT: computed tomography
DLBCL: diffuse large B-cell lymphoma
FDG: < Superscript>18</Superscript> F-fluorodeoxyglucose
mAb: monoclonal antibody
MRI: magnetic resonance imaging
PET: positron emission tomography
SUVavg: average standardized uptake value
