# Supplementary material for: CD19-immunoPET for noninvasive visualization of CD19 expression in B-cell lymphoma patients
Source: Biomark Res. 2024 May 12;12:50. doi: 10.1186/s40364-024-00595-9 (PMC11089670; doi:10.1186/s40364-024-00595-9)
Supplement: Supplementary file 1 — Supplementary Material 1 [file 40364_2024_595_MOESM1_ESM.docx]

**SupplementaRY FIGURES**


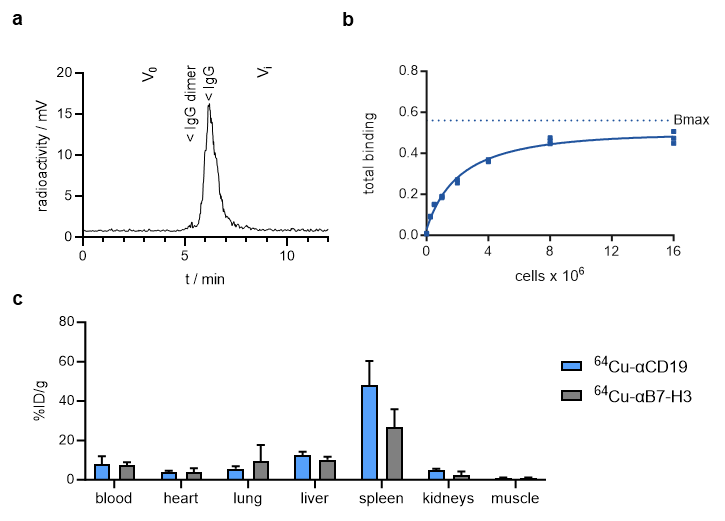


**FIGURE S1 Preclinical evaluation of ^64^Cu-αCD19 (a)** High-performance liquid chromatography (HPLC) after chelator conjugation with DOTA and radiolabeling with ^64^Cu. **(b)** Antigen excess binding assay to determine the maximum binding (Bmax) of ^64^Cu-αCD19, referred to as the immunoreactive fraction. Radiolabeled ^64^Cu-αCD19 (10 ng) was applied to an increasing number of Daudi cells (Triplicates, representative data out of three individual experiments, one site nonlinear regression). **(c)** *Ex vivo* organ biodistribution of ^64^Cu-αCD19 and ^64^Cu-B7H3 as measured by γ-counting.


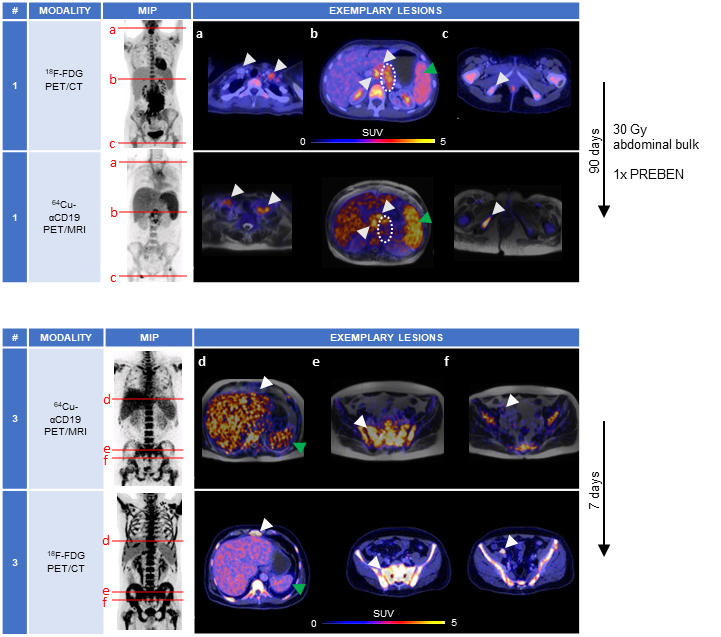


**FIGURE S2 Comparison of CD19-PET to ^18^F-FDG-PET.** Maximum intensity projection (MIP) of standardized uptake values (SUV) of CD19-PET and ^18^F-FDG-PET of patient 1 and 3. (**a-f**) Exemplary transversal images (levels are marked in the MIP by red lines) of intermodality-matched lymphoma lesions (indicated by white arrows) and spleen (green arrow) are shown for each patient. Irradiated lymphoma bulk of patient 1 (b) is marked in dotted white circle.

**SupplementaRY Tables**

**TABLE S1 Patient characteristics.** FL = follicular lymphoma; DLBCL = diffuse large B-cell lymphoma; MZL = marginal zone lymphoma; EPOCH = etoposide, prednisolone, vincristine, cyclophosphamide, anthracycline; R = rituximab; RTx = radiotherapy; per = peritoneal bulk; PREBEN = pixantrone, rituximab, etoposide, bendamustine; CHOP = cyclophosphamide, doxorubicine, vinciristine, prednisolone, BEAM = BCNU, etoposide, cytarabine, melphalan; autoSCT = autologous stem-cell transplantation.

| **Pat #** | **age** | **sex** | **histology** | **CD19 expression** | **stage** | **prior treatments** | **Rituximab prior PET** |
| --- | --- | --- | --- | --- | --- | --- | --- |
| 1 | 25 | F | FL, 3b | ++ | IV B | EPOCH-R, RTx (per), PREBEN | ongoing |
| 2 | 68 | F | DLBCL, from FL | +++ | II E | R-CHOP, R-BEAM, autoSCT, R, Idealisib | >6 mths |
| 3 | 58 | M | DLBCL | +++ | IV B | R-CHOEP, R-MTX-ifosphamide, AraC/Thiotepa, autoSCT, | 4.5 mths |
| 4 | 80 | M | MZL | + | IV B | R/bendamustine, R/chlorambucil, R-CHOP | <2 mths |

**SupplementaRY MethoDs**

**^64^Cu Production**

^64^Cu was produced at a PETtrace cyclotron (16.5 MeV proton energy; GE Medical Systems, Uppsala, Sweden) by proton irradiation of ^64^Ni electroplated on a platinum/iridium plate (90/10) (30 mg, > 98% enrichment; Chemotrade, Düsseldorf, Germany) and separated from metallic impurities by ion-exchange chromatography (AG^®^ 1-X8, Bio-Rad Laboratories, Hercules, CA, USA).

**Radiolabeling**

The radiopharmaceutical for human application was prepared under the direct responsibility of the nuclear medicine physician within the framework of article §13 2b of the German Pharmaceuticals Act (“Arzneimittelgesetz”; AMG) in a good manufacturing practice (GMP) facility. First, the anti-CD19 antibody (anti-human CD19 4G7, chimeric monoclonal antibody from CHO cells, GMP grade, Celonic AG, Basel, Switzerland) was conjugated using the chelator DOTA-NHS ester (2,2′,2”-(10-(2-((2,5-dioxopyrrolidin-1-yl)oxy)-2-oxoethyl)-1,4,7,10-tetraazacyclododecane-1,4,7-triyl)triacetic acid; CheMatech, Dijon, France).

All buffers were treated with 1.2 g/L Chelex 100 (sodium form, Sigma-Aldrich, St. Louis, MO, USA) to avoid contamination with metals. For removal of metal contaminations, the antibody was pretreated with 0.5 M EDTA for 30 min at room temperature (RT). Then, the buffer was changed to 0.1 M 4-(2-hydroxyethyl)-1-piperazineethanesulfonic acid (HEPES) pH 7.5. DOTA-NHS ester was dissolved in water for injection and added to the antibody (55-fold molar excess) followed by incubation at 4 °C for 12 h. After washing with 0.25 M sodium acetate pH 5.8, the conjugated antibody was characterized with respect to appearance (clear, colorless, no visible particles), protein concentration (result: 3.6 mg/mL), HPLC (SEC) profile, endotoxins, and radiolabeling properties (test synthesis). This conjugate can be stored for 5 years (2–8 °C) without significant loss in quality.

Radiolabeling was performed for individual patient application according to a modification of a procedure described in the literature ([1](#_ENREF_1)). Briefly, aliquots of chelator-conjugated antibody were incubated with a buffered solution of [^64^Cu]CuCl_2_ (2 µg/MBq) at 38–39 °C for 1 h. After radiolabeling, 1 µL of 20% Ca- diethylenetriaminepentaacetic acid (DTPA; Heyl, Berlin, Germany, injection solution for human use) was added to the tracer solution followed by dilution with phosphate buffered saline (PBS) and sterile filtration. The product obtained was tested according to pharmacopoeia methods (n = 6). The sterile, clear, colorless solution was free of visible particles and had a pH = 6.9–7.4. The endotoxin content was < 6.5 I.E./mL. Identity and chemical purity were confirmed using HPLC (SEC). Radiochemical purity (TLC, HPSEC) was > 95%, and radionuclide purity was >99.9%. The specific radioactivity was ca. 0.5 MBq/µg of conjugated antibody.

**Cell Lines**

The human B lymphoblast cell line Daudi, obtained from ATCC (Manassas, VA, USA), was cultured in RPMI-1640 medium supplemented with 1% penicillin/streptomycin and 10% fetal calf serum (FCS; Biochrom GmbH, Berlin, Germany) at 37°C and 5% CO_2_.

***In Vitro* Binding Assay**

The immunoreactive fraction was quantified by calculating the maximum binding (B_max,_ nonlinear regression) with increasing numbers of Daudi cells incubated with a constant quantity of mAb (10 ng) for 1 h at 37°C. After two washing steps with PBS/1% FCS, the remaining cell-bound radioactivity was measured using a Wizard² 2480 gamma counter (PerkinElmer, Inc., Waltham, MA, USA) and quantified as a percentage of the total added activity.

**Mouse Model**

Seven-week-old female CD-1 nude (Crl:CD1-*Foxn1^nu^*) were purchased from Charles River Laboratories (Sulzfeld, Germany) and kept under standardized conditions. All the experiments were carried out in accordance with the German Animal Welfare Act and with the consent of regulatory authorities (Regierungspräsidium Tübingen).

CD-1 nude mice were irradiated with 6 Gy one day prior to tumor cell injection. For tumor inoculation, 6 x 10^6^ Daudi cells were resuspended in 50% Matrigel (BD) and 50% PBS and were then subcutaneously (*s.c.*) injected into the right flanks of CD1 nude mice.

**Preclinical PET/MR Imaging**

Radiolabeled mAbs (~12 MBq/50 μg) were injected intravenously (*i.v.)* when tumors reached a size of approximately 1 cm^3^. Simultaneous PET/MRI scans were performed on a small animal 7 T ClinScan magnetic resonance scanner (Bruker BioSpin GmbH, Rheinstetten, Germany) with an installed PET insert developed at our laboratory as previously described ([2](#_ENREF_2)). Anatomical T2 TurboRARE images and static PET images with a 10 min acquisition time were acquired at the indicated time points. PET images were reconstructed using an ordered subset expectation maximization (OSEM-2D) algorithm and analyzed with Inveon Research Workplace (Siemens Preclinical Solutions). The volume of interest (VOI) in each organ was defined based on anatomical MRI data used to determine the corresponding PET tracer uptake. The resulting values were decay-corrected. *Ex vivo* γ-counting was conducted immediately after the last imaging time point by measuring the weight and radioactivity of the organs of interest.

**Generation of CAR T cells**

The applied αCD19 CAR is based on a scFv derived from the αCD19-mAb 4G7 assembled on a second-generation CAR backbone incorporating a CD8 hinge, CD8 transmembrane domain, and the cytoplasmic domain of 41BB and CD3zeta ([3](#_ENREF_3), [4](#_ENREF_4)). Lentivirus (LV) for T-cell transduction was produced in Lenti-XTM 293T (TaKaRa Bio, Kusatsu, Japan) after 3 plasmid transfections using Lipofectamine 3000 (Thermo Fisher) of the second-generation packaging plasmid psPAX2 (Addgene), VSV-G envelope plasmid pMD2.G (Addgene) and the CAR-containing transfer plasmid. For CAR-T-cell production, PBMCs were isolated from whole peripheral blood acquired from healthy volunteer donors at the University Children’s Hospital Tuebingen by Ficoll-Paque density gradient centrifugation (Biocoll, Biochrom, Berlin, Germany). T cells were isolated using CD4 and CD8 microbeads (Miltenyi Biotec). T cells were activated with TransActTM (anti-CD3 and anti-CD28 agonistic signals, Miltenyi Biotec) and cultivated in TexMACS media (Miltenyi Biotec) supplemented with 10 ng/mL IL-7 and 5 ng/mL IL-15 (Miltenyi Biotec). After 24 h, activated T cells were transduced at a multiplicity of infection (MOI) of 3. Transduced T cells were maintained at 0.5–2 × 10^6^ cells/mL in IL7/IL15 containing TexMACS® media.

**Luciferase-based cytotoxicity assay (LCA)**

Tumor cells were plated in RPMI 1640-based complete media (see above) at 50,000 cells per 96 flat bottom wells in white plates. Synthetic D-luciferin (Sigma Aldrich) was added at 4 µg/mL. αCD19-CAR T cells were plated at the indicated effector-to-target ratio. The total volume per well was 200 µl. The αCD19-mAb 4G7SDIE was added at the indicated concentrations. Plates were incubated in a HERA cell incubator (Heraeus, Hanau, Germany) at 37 °C, 95% humidity, and 5% CO_2_. Plates were measured using the Wallac Victor 1420 Multilabel Counter (Perkin Elmer) at 37 °C at the indicated time intervals. Lysis was determined by the relative luminescence of the testing condition to the dilution series according to the standard controls.

**Patient Selection and Clinical PET/MR Imaging**

Patients with B-NHL were individually selected by their referring physicians with immunohistochemical confirmed CD19-positivity to evaluate the eligibility for CD19-directed therapy. Simultaneous PET/MR data were acquired 19-25 h after *i.v.* injection of 203±57 MBq ^64^Cu-αCD19 (0.5 MBq/µg) on an integrated 3T whole-body PET/MRI system (Biograph mMR, Siemens Healthineers, Erlangen, Germany). T1-weighted, 3D-encoded spoiled gradient-echo with double-echo for Dixon-based fat-water separation for attenuation correction as well as coronal/axial T2 HASTE and axial T2 TIRM MR sequences were performed. PET images were reconstructed using an OSEM-3D algorithm corrected for scatter and attenuation based on standard MRI-based segmentation as previously described ([5](#_ENREF_5)). For semiquantitative PET analysis, representative regions of interest with 40% isocontour were defined by dedicated software packages (Syngo.via, Siemens Healthineers, Erlangen and Affinity Viewer, Hermes Medical Solutions, Stockholm, Sweden).

**Statistical Analyses**

The data were analyzed using GraphPad Prism, version 10 (GraphPad Software, Inc., San Diego, CA, USA). The values are expressed as the arithmetic means ± standard deviations (SD) unless otherwise indicated. For statistical analyses, unpaired t tests were applied. Binding curves were analyzed using a one-site nonlinear regression model. A value of p < 0.05 was considered to indicate statistical significance and is marked as * for p < 0.05, ** for p < 0.01, *** for p < 0.001, and **** for p < 0.0001.

**REFERENCES**

1. Cooper MS, Sabbah E, Mather SJ. Conjugation of chelating agents to proteins and radiolabeling with trivalent metallic isotopes. Nature protocols. 2006;1(1):314-7.

2. Wehrl HF, Hossain M, Lankes K, Liu C-C, Bezrukov I, Martirosian P, et al. Simultaneous PET-MRI reveals brain function in activated and resting state on metabolic, hemodynamic and multiple temporal scales. Nature medicine. 2013;19(9):1184-9.

3. Seitz CM, Schroeder S, Knopf P, Krahl A-C, Hau J, Schleicher S, et al. GD2-targeted chimeric antigen receptor T cells prevent metastasis formation by elimination of breast cancer stem-like cells. Oncoimmunology. 2020;9(1):1683345.

4. Seitz CM, Mittelstaet J, Atar D, Hau J, Reiter S, Illi C, et al. Novel adapter CAR-T cell technology for precisely controllable multiplex cancer targeting. Oncoimmunology. 2021;10(1):2003532.

5. Hofmann M, Bezrukov I, Mantlik F, Aschoff P, Steinke F, Beyer T, et al. MRI-based attenuation correction for whole-body PET/MRI: quantitative evaluation of segmentation- and atlas-based methods. Journal of nuclear medicine : official publication, Society of Nuclear Medicine. 2011;52(9):1392-9.
